# PIG7 promotes leukemia cell chemosensitivity via lysosomal membrane permeabilization

**DOI:** 10.18632/oncotarget.6739

**Published:** 2015-12-23

**Authors:** Jiazhuo Liu, Leiwen Peng, Ting Niu, Yu Wu, Jianjun Li, Fangfang Wang, Yuhuan Zheng, Ting Liu

**Affiliations:** ^1^ Department of Hematology, West China Hospital, Sichuan University, Chengdu, Sichuan 610041, China; ^2^ Department of Laboratory Medicine, West China Second University Hospital, Sichuan University, Chengdu, Sichuan 610041, China

**Keywords:** PIG7, LMP, chemosensitivity, cathepsin, ROS

## Abstract

PIG7 localizes to lysosomal membrane in leukemia cells. Our previous work has shown that transduction of pig7 into a series of leukemia cell lines did not result in either apoptosis or differentiation of most tested cell lines. Interestingly, it did significantly sensitize these cell lines to chemotherapeutic drugs. Here, we further investigated the mechanism underlying pig7-induced improved sensitivity of acute leukemia cells to chemotherapy. Our results demonstrated that the sensitization effect driven by exogenous pig7 was more effective in drug-resistant leukemia cell lines which had lower endogenous pig7 expression. Overexpression of pig7 did not directly activate the caspase apoptotic pathway, but decreased the lysosomal stability. The expression of pig7 resulted in lysosomal membrane permeabilization (LMP) and lysosomal protease (e.g. cathepsin B, D, L) release. Moreover, we also observed increased reactive oxygen species (ROS) and decreased mitochondrial membrane potential (ΔΨm) induced by pig7. Some autophagy markers such as LC3I/II, ATG5 and Beclin-1, and necroptosis maker MLKL were also stimulated. However, intrinsic antagonism such as serine/cysteine protease inhibitors Spi2A and Cystatin C prevented downstream effectors from triggering leukemia cells, which were only on the “verge of apoptosis”. When combined with chemotherapy, LMP increased and more proteases were released. Once this process was beyond the limit of intrinsic antagonism, it induced programmed cell death cooperatively via caspase-independent and caspase-dependent pathways.

## INTRODUCTION

Pig7 (P53 induced gene 7) was initially believed to be one of the key genes involved in P53-induced apoptosis, serving as a bridge between P53 expression and apoptosis [1]. It was later found that Pig7 had two completely different transcripts: LITAF (LPS-Induced TNF Alpha Factor) and SIMPLE (Small Integral Membrane Protein of Lysosome/late Endosome). LITAF acts as a transcriptional promoter in LPS-induced TNF alpha expression [2, 3], and SIMPLE functions as an intrinsic protein in the early endosomal/lysosomal membrane. However, its functions within these membranes are not clearly understood [4, 5]. In a previous study, we used PCR and restriction endonucleases to identify transcripts in ten leukemia cell lines (K562, HL60, U937, NB4, Kasumi-1, SKNO-1, KG1a, Nalm-6, Jurkat and Raji) as well as primary cells derived from the bone marrow of acute leukemia patients [6]. The results suggest that the SIMPLE transcript was the only one present, while the expression of LITAF was undetectable. Moreover, SIMPLE expression in leukemia cells was found to be lower than in healthy cells. When these leukemia cells were treated with chemotherapeutic drugs (e.g. PB, ATRA, and VP16), we found a significant increase (22.5–27.8 times) in endogenous expression of pig7 during cellular apoptosis. These results indicated that pig7 may be closely related to cellular apoptosis. When pig7 was transfected into K562, HL60, NB4, Raji, Kasumi-1 and SKNO-1 cells, we found that for most leukemia cells, overexpression of pig7 did not directly affect either their proliferation or differentiation. Furthermore, any pig7-induced promotion of apoptosis was also not evident. However, when combined with chemotherapies such as using VP16 and daunorubicin, we found that there was a significant enhancement in the drug sensitivity of the tested leukemia cells [6, 7].

In recent years, increasing evidence has shown that pig7 can inhibit tumor growth and that the causal mechanism may be involved in the regulation of cellular apoptosis [8–10]. As we have confirmed that the only pig7 transcript present in leukemia cells is the SIMPLE transcript—which is localized in the lysosomal membrane—and we speculated that the chemosensitivity promoting effects of pig7 may be associated with the lysosome.

In the present study, we transfected pig7 into two kinds of leukemia cell lines (K562, HL60), two kinds of drug-resistant leukemia cell lines (K562/ADM, and HL60/ADM), and five cases of acute myeloid leukemia primary cells. We found that the sensitization effect driven by exogenous pig7 was more effective in K562/ADM and HL60/ADM which had lower endogenous pig7 expression compared with other cells. High expression of exogenous PIG7 induced lysosomal membrane permeabilization (LMP) and PIG7 translocated into the cytosol and proteases (e.g. cathepsins) were subsequently released into the cytosol. Meanwhile, the levels of reactive oxygen species (ROS) and mitochondrial membrane potential (ΔΨm) were found to be significantly changed. Expressions of autophagy markers such as LC3I/II, ATG5 and Beclin-1 and necroptosis marker MLKL also increased. Interestingly, in parallel to alteration of these cell death signals, neither apoptosis was present nor did the expression of caspase show any obvious changes. So we further searched intrinsic antagonisms which prevented downstream effectors from triggering death of leukemia cells, and we found with the release of cathepsins into the cytosol, the endogenous expression of serine/cysteine protease inhibitors such as Spi2A and Cystatin C increased simultaneously. That may be an intrinsic antagonism which protected leukemia cells. However, when combined with chemotherapy, LMP significantly increased; more proteases were released, and more ROS were formed. When beyond the limit of intrinsic antagonism, PIG7 can induce cellular apoptosis and necroptosis. In addition, cathepsin inhibitors (e.g. CA-074Me, pepstatin A and E64D) and antioxidants (e.g. N-acetylcysteine and α-toco) both provided significant protection on cells from programmed cell death by the combination of PIG7 and chemotherapy. Further, the caspase inhibitor Z-VAD-FMK and necroptosis inhibitor Necrosulfonamide (NSA) did not afford the same protection when used alone, but could have a significant protective effect when used in combination, confirming caspase-dependent and -independent cell death pathways were both involved in this process.

## RESULTS

### Endogenous pig7 expression levels are related to its chemosensitivity-promoting effects

**Figure 1:**
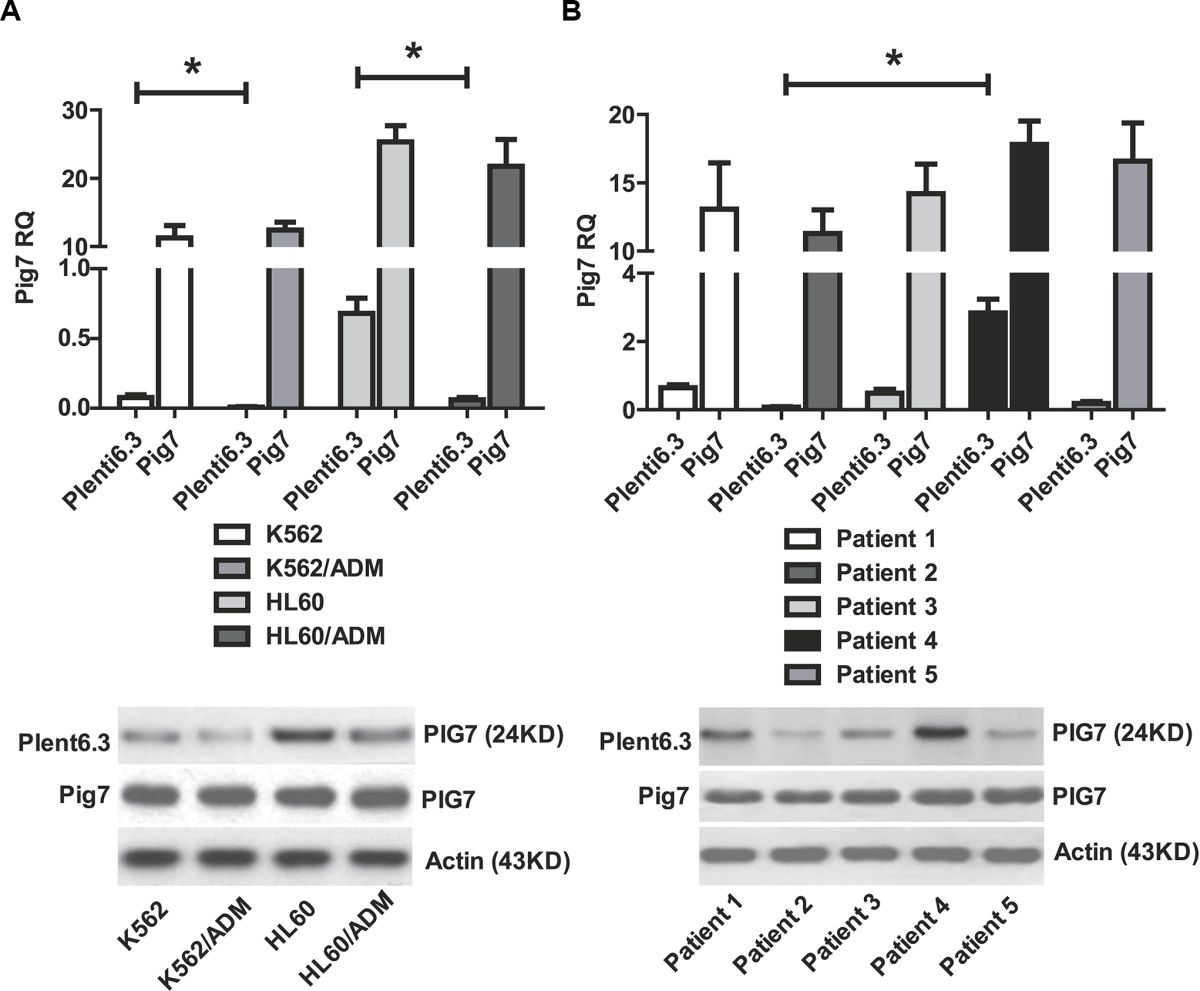
Expression of pig7 mediated by lentivirus infection (**A**) Endogenous expression of pig7 in K562/ADM and HL60/ADM cell lines was significantly lower than in K562 and HL60 cell lines (*P < 0.01). (**B**) Patient 2 had the lowest expression of endogenous pig7 and Patient 4 had the highest expression (*P < 0.001). In all cells, high levels of pig7 product could be detected in the plent6.3-PIG7 group by RT-PCR and Western blot at 48 h post-lentiviral infection. There was no significant difference in pig7 protein expression (*P* < 0.05).

**Figure 2:**
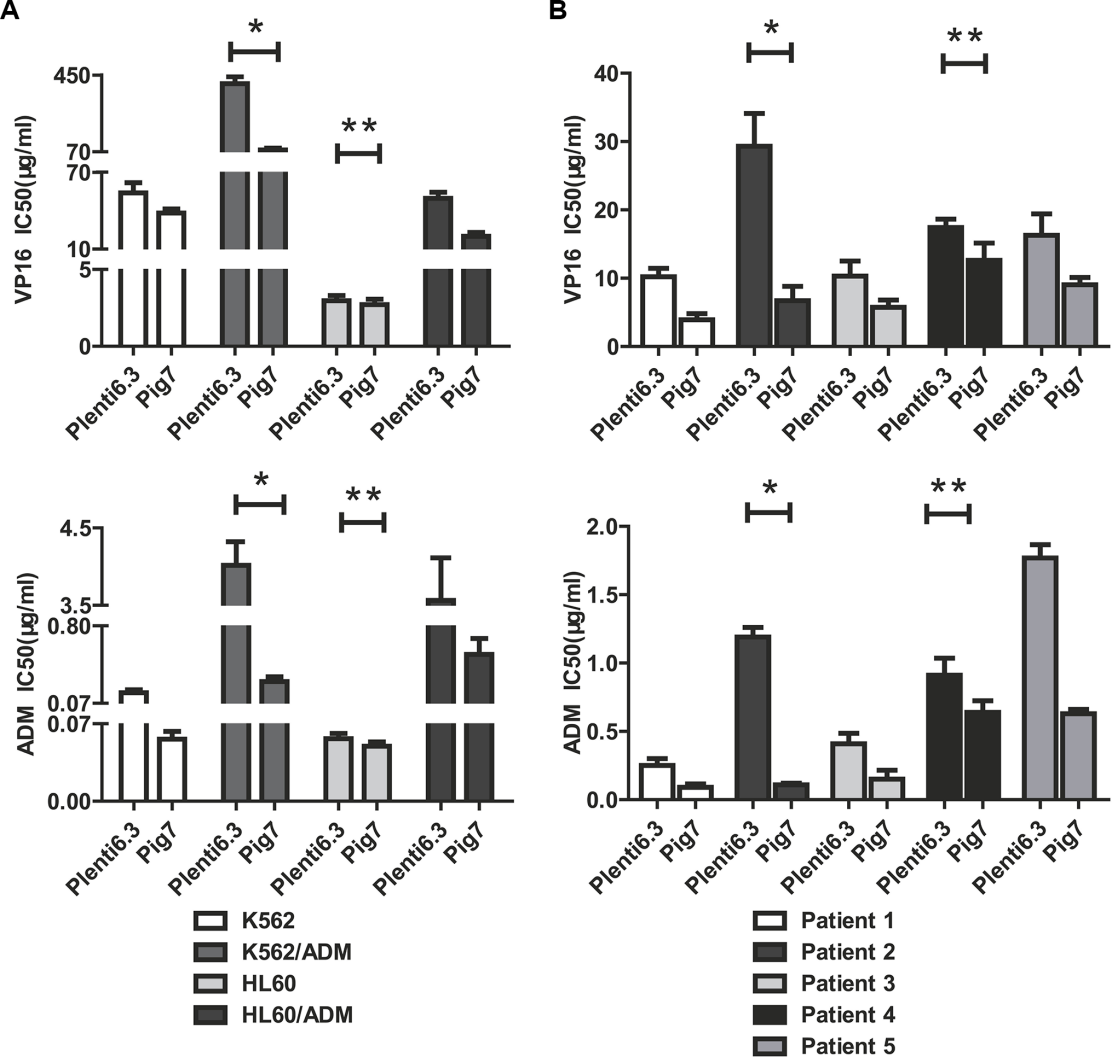
MTT assay and decreased IC_50_ in cells infected for 48 h with Plent6.3-PIG7 in combination with either VP16 or ADM treatment (**A**) The IC_50_ of VP16 and ADM for K562/ADM cells decreased by 5.1- and 14.3-fold, respectively (*). In HL60 cells, IC_50_ did not significantly change (**P > 0.05). (**B**) In the five cases of primary cells, the IC_50_ of VP16 and ADM for Patient 2 at 48 h post-infection decreased by 4.3- and 9.9-fold (*), respectively. In Patient 2, the IC_50_ only decreased to 1.3- and 1.6-fold, respectively (**).

**Figure 3:**
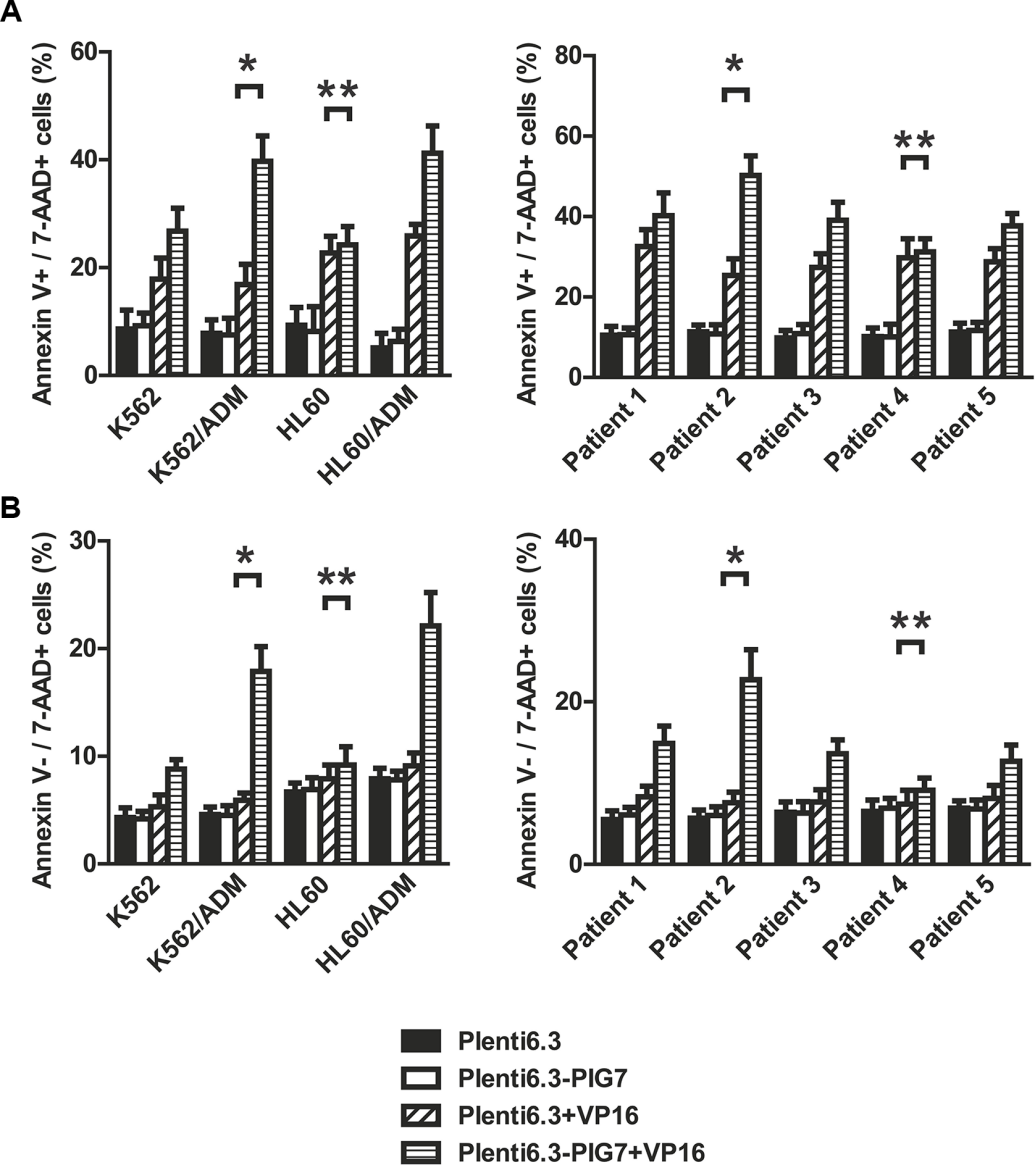
Changes in apoptosis and necroptosis of leukemia cells after lentiviral infection and VP16 treatment (48 h) Annexin V staining assay indicated that (**A**) the apoptosis rates of these four cell lines and five cases of primary cells infected with Plent6.3-PIG7 lentivirus were not changed. When combined with differential concentrations of VP16, the apoptotic rate of these three cell lines and four cases of primary cells significantly increased when compared with Plent6.3 control + VP16 groups. K562/ADM and Patient 2 has the highest increase in apoptosis rate (*). There was almost no change in apoptosis rate in either HL60 or Patient 4 cells (**P > 0.05) (**B**) The necroptosis rate of cells infected with Plent6.3-PIG7 lentivirus was unchanged. When combined with VP16, the necroptosis rate of all cells increased compared with Plent6.3 control + VP16 groups. K562/ADM and Patient 2 had the highest increases in their respective necroptosis rates (*), while HL60 and Patient 4 had the lowest increases in their necroptosis rates (**).

Previous studies from our group have shown that for most leukemia cells (e.g. K562, NB4, Kasumi-1, and SKNO-1), overexpression of pig7 can enhance their chemotherapeutic sensitivity [6, 7]. In order to explore the mechanism behind this effect, we selected two ordinary leukemia cell lines (K562 and HL60), two chemotherapy resistant cell lines (K562/ADM, HL60/ADM), and primary bone marrow mononuclear cells from five acute myeloid leukemia patients. We then transduced pig7 into these cells via lentiviral delivery, using an experimental design featuring a pig7 transduced group (Plent6.3-PIG7 group) and an empty viral vector control group (Plent6.3 group). At 48 h post-infection, the expression level of pig7 mRNA was measured using real-time quantitative PCR and the expression of PIG7 protein was detected using Western blot. Changes in chemosensitivity were analyzed by MTT assay and Annexin V staining. Strikingly, both endogenous pig7 mRNA and protein expression levels were decreased (*P < 0.001) (Figure 1A) in chemotherapy resistant cell lines (K562/ADM and HL60/ADM). Among the primary cells, Cells from patient 2 had the lowest expression of endogenous pig7 while those from patient 4 had the highest expression (*P < 0.001) (Figure 1B). After transfection with lentivirus Plenti6.3-PIG7, the mRNA and protein expressions of pig7 were both significantly increased, reaching very high levels in all cells. However, protein expression of pig7 showed no significant differences in either the four kinds of cell lines or in the five cases of primary cells. Overexpression of pig7 disproportionately enhanced the chemosensitivity of these cells, with the exception of the HL60 cell line. Among the four cell lines, the IC_50_ values of VP16 and ADM at 48 h for K562/ADM cells, which had the lowest expression of endogenous pig7, were reduced from 407.3 μg/ml and 4.01 μg/ml for the Plent6.3 group to 79.6 μg/ml and 0.28 μg/ml for the Pig7 groups, respectively. Their chemosensitivity also increased 5.1- and 14.3-fold, respectively. HL60 cells had a relatively high endogenous expression of pig7 and the 48 h IC_50_ values of both VP16 and ADM were not significantly changed (**P > 0.05) (Figure 2A). In the five cases of primary cells, patient 2 had the lowest expression of endogenous pig7 and also had decreased IC_50_ at 48 h for both VP16 and ADM (from 29.3 μg/ml and 1.19 μg/ml to 6.7 μg/ml and 0.12 μg/ml, respectively). Their chemosensitivity increased 4.3- and 9.9-fold, respectively. In contrast to patient 2, patient 4 had the highest expression of endogenous pig7 and did not have significant changes in IC50 of either VP16 or ADM at 48 h. Their chemosensitivity only increased 1.3- and 1.6-fold, respectively (Figure 2B). Annexin V staining assay indicated that the largest increase in the apoptosis rate (Annexin V+/7-AAD+ cells%) occurred in K562/ADM and patient 2 primary cells treated with both Plent6.3-PIG7 and VP16 (39.7 ± 4.7% VS 16.9 ± 3.9%, 50.2 ± 4.8% VS 25.4 ± 3.1%, respectively, *P < 0.01) (Figure 3A). The necroptosis rate increase (Annexin V−/7-AAD+ cells%) of these cells was also the highest (17.9 ± 2.3% VS 5.9 ± 0.7%, 22.7 ± 3.7% VS 7.6 ± 1.3%, respectively, *P < 0.01) (Figure 3B). However, in HL60 and patient 4 primary cells, the apoptosis rate was not significantly changed (24.2 ± 3.4% VS 22.7 ± 3.1%, 31.2 ± 3.3% VS 29.8 ± 4.1%, respectively, **P > 0.05) (Figure 3A). The increase in the necroptosis rate in these cells was also very mild (10.2 ± 1.7% VS 7.9 ± 1.3%, 9.1 ± 1.5% VS 7.4 ± 1.7%, respectively, **P < 0.05) (Figure 3B). Collectively, these results indicate that the chemosensitivity promoting effect of pig7 is widely varied in both different leukemia cell lines and primary cells. Moreover, the expression level of endogenous pig7 may have a strong negative correlation with this observed chemosensitive effect.

### Overexpression of pig7 induces lysosomal membrane permeabilization (LMP) and cytosolic cathepsin release

Our previous study demonstrated that PIG7 (SIMPLE) localized to the lysosomal membrane in leukemia cells [6]. We thus speculated that the chemosensitivity promoting effect observed in this study may be associated with the lysosomal pathway. We chose two chemotherapy resistant cell lines and primary cells derived from Patient 2 as *in vitro* models and transduced pig7 into these cells by lentiviral delivery. Our experimental design included a pig7 transduced group (Plent6.3-PIG7 group), a group with a combination of pig7 virus and VP16 (Plent6.3-PIG7 + VP16 group), an empty virus group (Plent6.3 group), and a group with a combination of empty virus and VP16 (Plent6.3-PIG7 + VP16 group). At 48 and 72 h after infection, cells were stained with the lysosomotropic agent AO and LMP was monitored (i) qualitatively by fluorescence microscopy and (ii) quantitatively by flow cytometry. In both approaches, by measuring the fluorescence intensity of the cells, with increased green fluorescence in the cytosol indicating increased LMP. Since AO is a weak base, it can move freely across membranes when uncharged and accumulate in acidic compartments like lysosomes in its protonated form. In these acidic compartments, it then forms and aggregates that fluoresce bright red. LMP can be monitored due to its metachromatic emission spectrum, as an increase in diffuse cytosolic green fluorescence occurs with a concomitant decrease in lysosomal red fluorescence [11]. In our study, fluorescence microscopic analysis of cells with AO revealed that a high percentage of the Plent6.3 group (empty virus control) exhibited intact lysosomes (or, that the high levels of red fluorescence corresponded to accumulation of AO within the acidic lysosomes). Moreover, there was a reduced percentage of the Plent6.3-PIG7 group with diffuse green fluorescence (corresponding to non-lysosomal AO).

**Figure 4:**
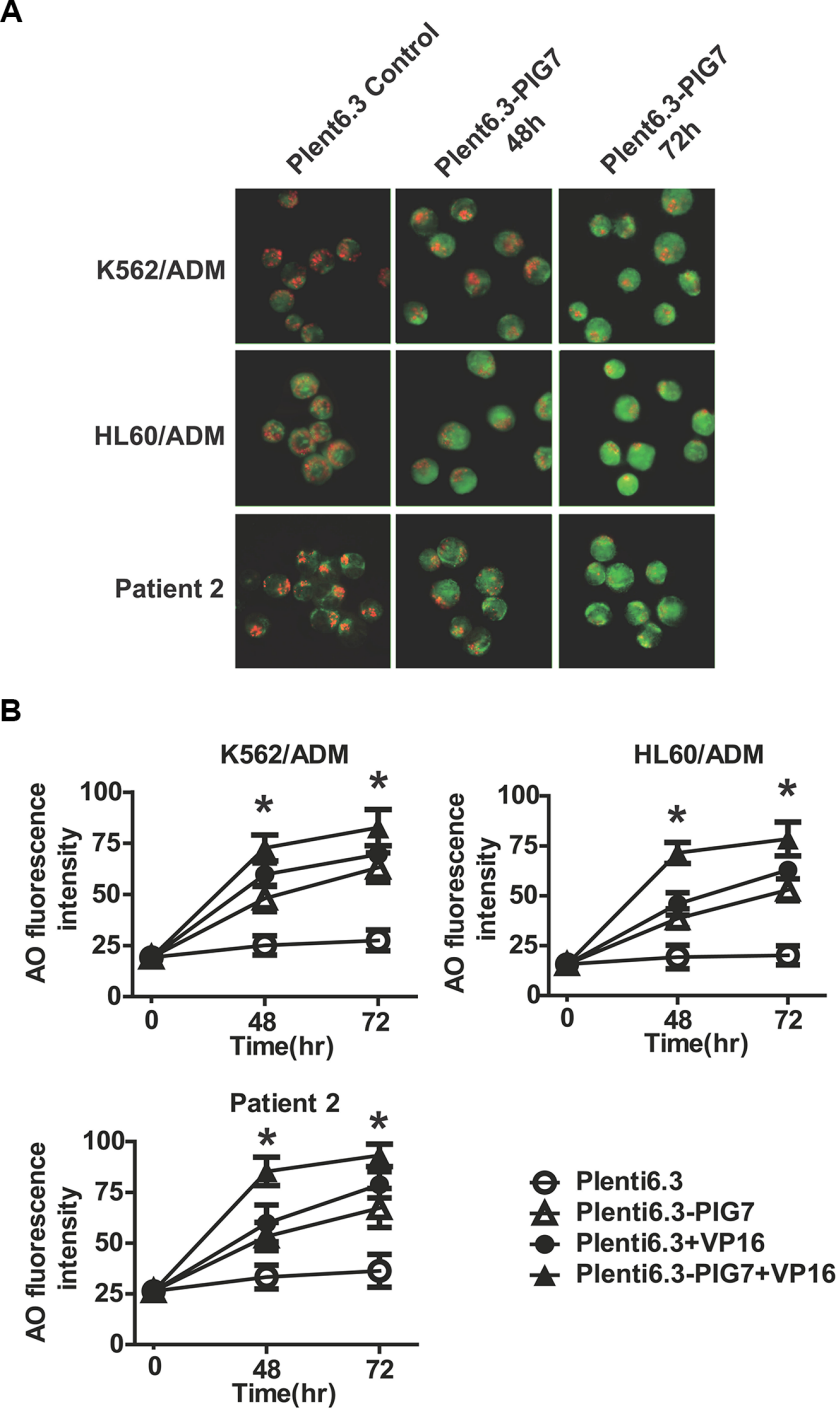
Overexpression of pig7 induces LMP (**A**) LMP detected by AO staining and visualized by fluorescence microscopy. (**B**) Percentage of leukemia cells displaying LMP quantified by flow cytometric analysis of AO staining after lentiviral infection in combination with VP16. 48 and 72 h green fluorescence intensities both significantly increased in the Plent6.3-PIG7 group. The Plent6.3-PIG7 + VP16 group had the highest green fluorescence intensity (*P < 0.05).

Contrastingly, overexpression of pig7 led to a decrease in the percentage of cells with red fluorescence and an associated increase in the percentage of cells with green fluorescence. These parallel findings are indicative of LMP (Figure 4A). Quantification of AO-stained cells was performed by flow cytometry. AO green fluorescence intensity of Plent6.3-PIG7 group was higher than the empty virus control group. At 72 h post-infection, the green fluorescence intensity of K562/ADM, HL60/ADM, and patient 2 primary cells increased from approximately 27.5, 20.1, and 36.3 in the Plent6.3 groups to 63.1, 52.9, and 67.2 in the Plent6.3-PIG7 groups, respectively. The combination of VP16 contributed to further lysosomal destabilization and aggravated LMP, resulting in the Plent6.3-PIG7 + VP16 group with the highest green fluorescence intensity (*P < 0.05) (Figure 4B).

**Figure 5:**
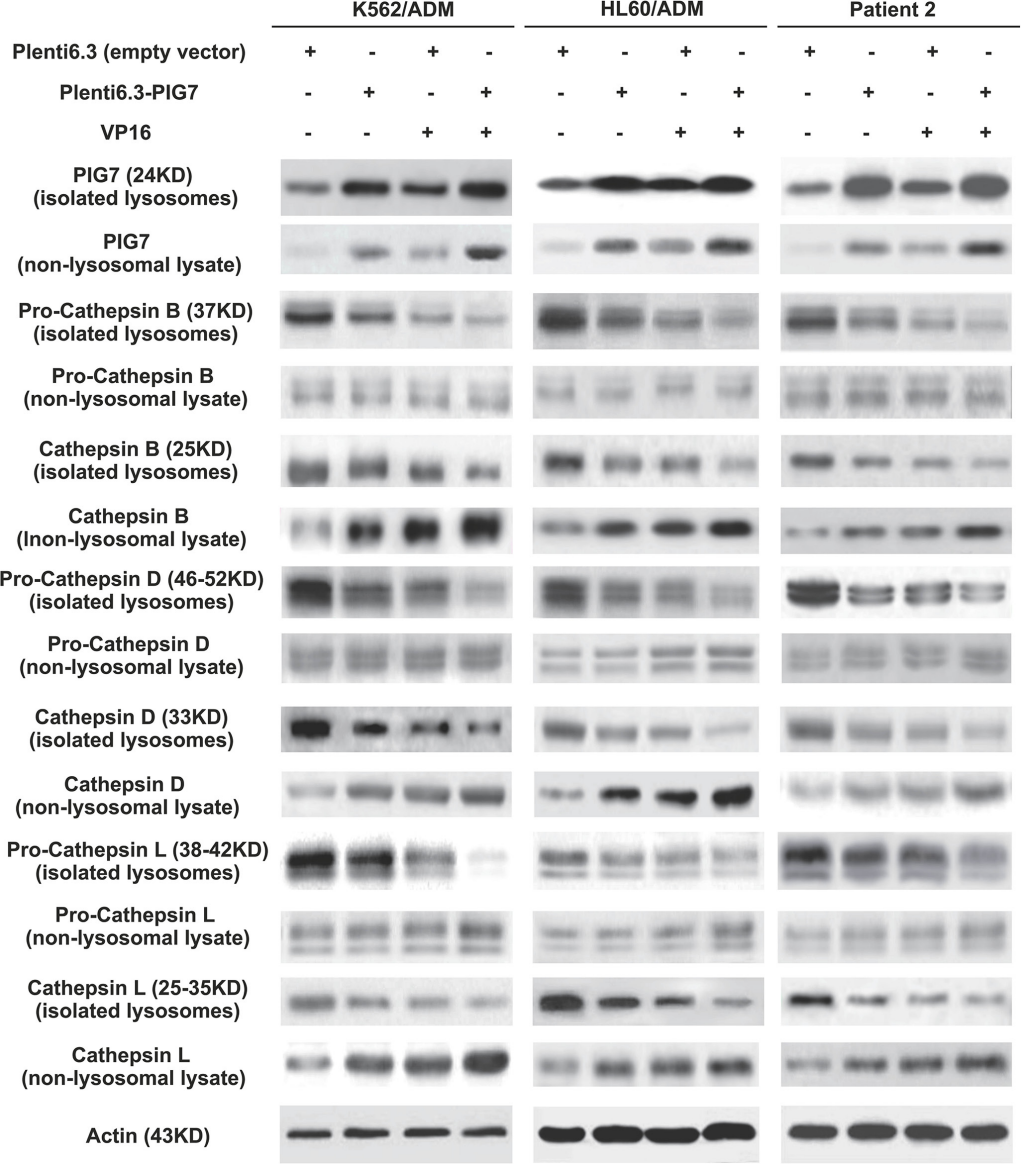
Effect of pig7 on the expression and cytosolic release of cathepsins in leukemia cells, when comparing isolated lysosomes and non-lysosome-containing lysate Overexpression of pig7 resulted in: **(i)** active cathepsin B, D and L release from lysosomes, **(ii)** increased precursors pro-cathepsin in B, D, and L were cleaved to their active forms, but fewer precursors were released into the cytosol, and **(iii)** the combination of VP16 further enhanced the above effects.

After LMP, lysosomal proteases such as cathepsins are released from lysosomes into the cytoso [12]. As such, their detection in the cytosolic fraction is indicative of LMP. We therefore collected treated cells at 72 h and isolated lysosomes using a commercially available Lysosome Enrichment Kit. The isolated lysosomes as well as the non-lysosomal lysate were collected for subsequent Western blot analysis. We found that in the Plent6.3 group, the activated Cathepsin B, Cathepsin D, and Cathepsin L proteins and their precursors (pro-cathepsin B, D, L) were mainly expressed in lysosomes and exhibited very low expression levels in the cytosol. Notably, we found that overexpression of pig7 led these activated cathepsins to be released into the cytosol, indicating that it induced LMP. We also observed that the combination of VP16 further aggravated LMP, yielding the highest level of activated cathepsin expression in the Plent6.3-PIG7 + VP16 group (Figure 5).

### Cathepsin inhibitors and antioxidants protect against cellular toxicity, but not caspase inhibition

**Figure 6:**
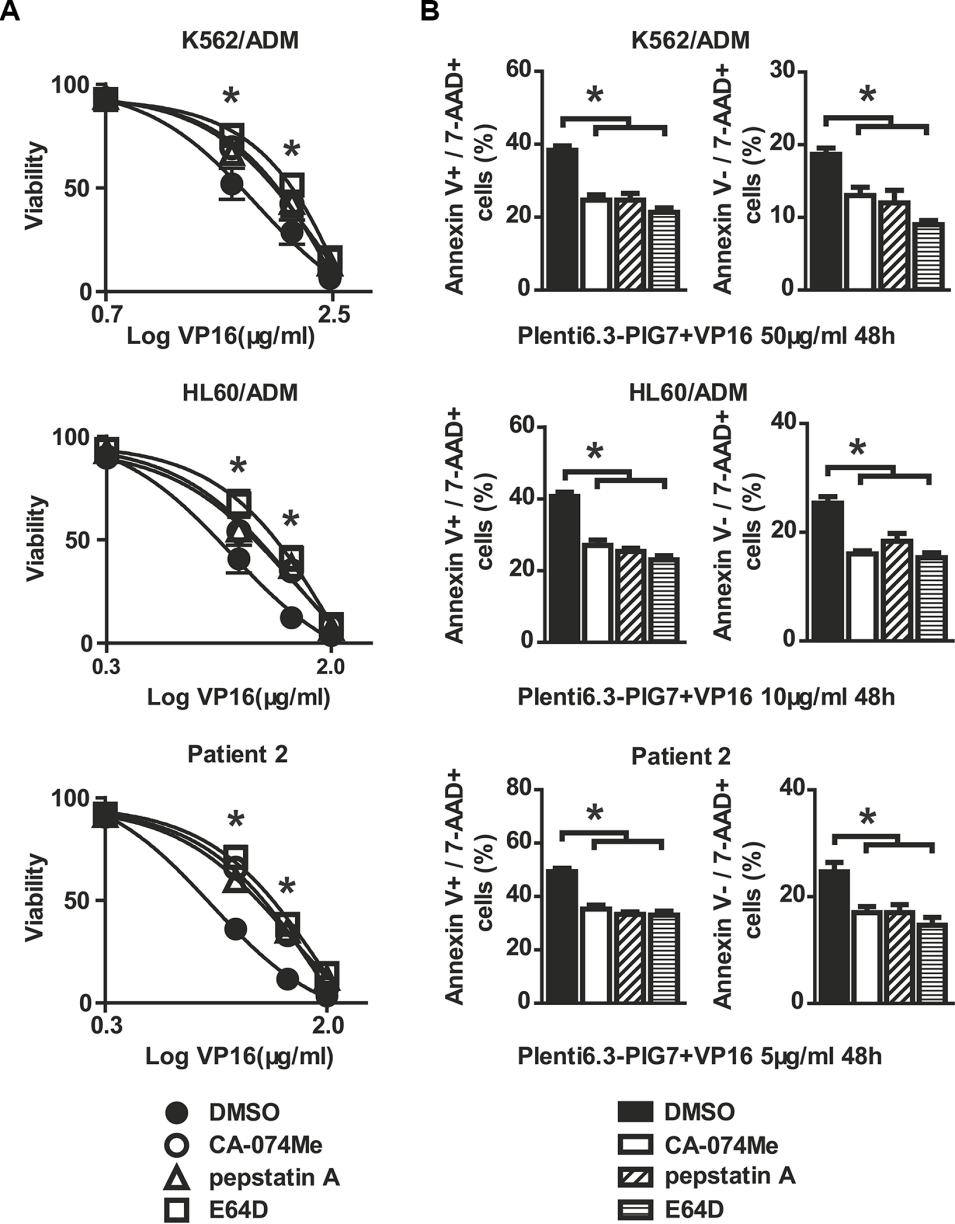
Cathepsin inhibitors are protective against cellular toxicity Viability and apoptosis/necroptosis rates of transfected cells following 48 h treatment with VP16 in the presence of CA-074-Me, pepstatin A, or E64D. Data represent percent viability and apoptosis/necroptosis rate when compared to DMSO-treated cells. (**A**) MTT assay indicated that three inhibitors increased the viability of leukemia cells in combination with Plent6.3-PIG7 and VP16 (*P < 0.05). (**B**) Annexin V staining also indicated that the three inhibitors protected leukemia cells from apoptosis and necroptosis (*P < 0.05).

To test whether the observed chemosensitivity-promoting effect was cathepsin-dependent, we performed selective inhibition of lysosomal cathepsins with CA-074Me (cathepsin B inhibitor), pepstatin A (cathepsin D inhibitor) and E64D (cathepsins B and L inhibitor). We also chose the previously used cell lines along with the following experimental groups: CA-074Me, pepstatin A, E64D, and DMSO as vehicle control. We then examined the resulting changes in chemosensitivity (VP16) at 48 h post-infection with the Plenti6.3-PIG7 virus using both the MTT assay and Annexin V staining. We found that incubation with CA-074Me, Pepstatin A, or E64D all individually and significantly (*P < 0.05) increased cellular viability (Figure 6A). Annexin V staining also indicated that these three inhibitors had obvious antagonistic effects on both apoptosis and necroptosis (*P < 0.05 for both) (Figure 6B).

**Figure 7:**
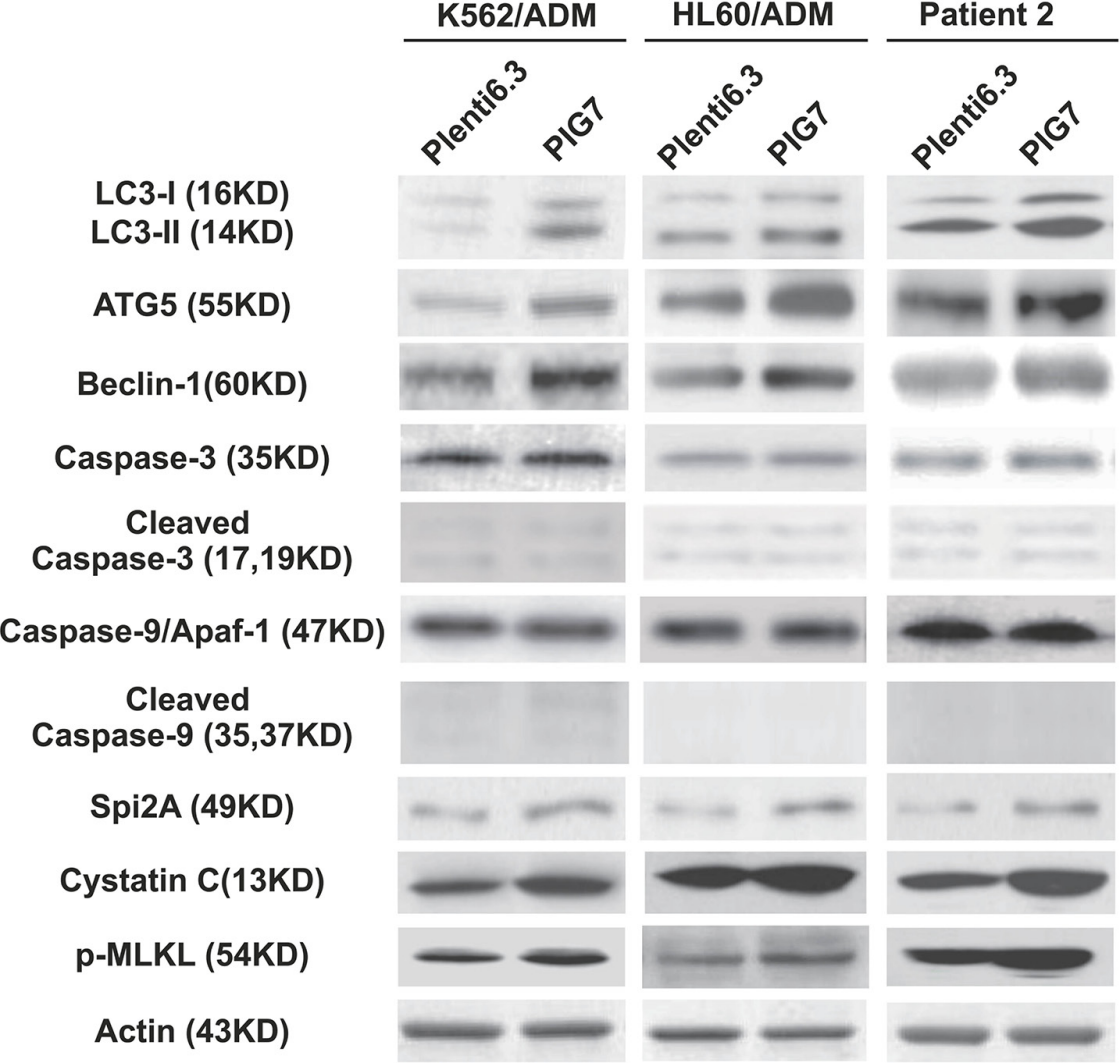
Effect of pig7 on the expression of markers for autophagy, caspases, intrinsic protease inhibitors, and necroptosis in leukemia cells Higher levels of autophagy markers (LC3I/II, ATG5 and Beclin-1), serine/cysteine protease inhibitors (Spi2A and Cystatin C), and necroptosis marker (p-MLKL) product were detected in the Plenti6.3–PIG7 group. Active forms for both caspase 9 and caspase 3 did not increase.

**Figure 8:**
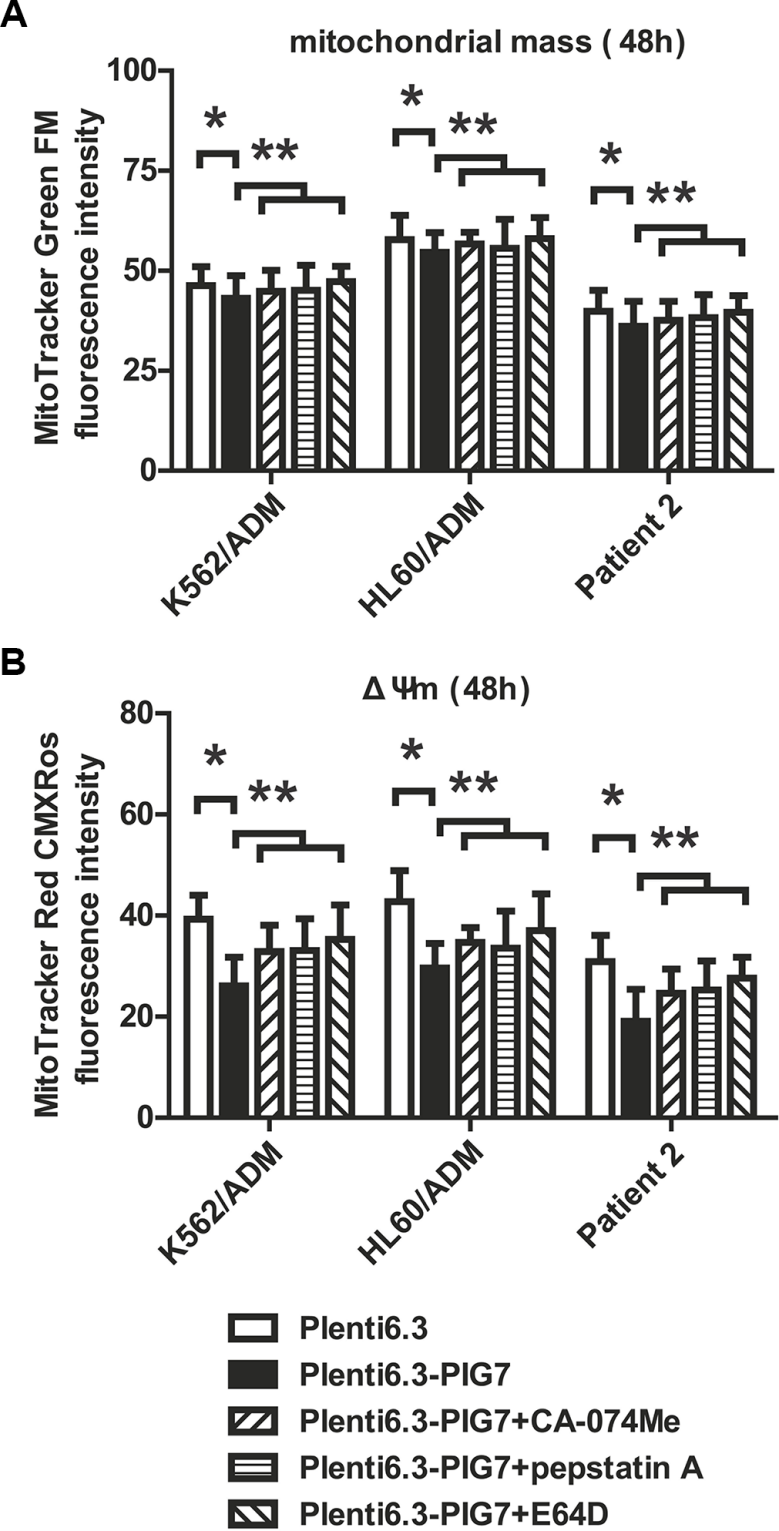
Effect of pig7 on the changes in mitochondrial mass and membrane potential (ΔΨm) (**A**) Almost no change in mitochondrial mass was observed in the Plenti6.3–PIG7 group (*p > 0.05), which was not improved in the presence of cathepsin inhibitors (CA-074-Me, pepstatin A, or E64D) (**p > 0.05) (**B**) Decreased ΔΨm was detected in the Plenti6.3–PIG7 group (*p < 0.05) and it was reversed in the presence of cathepsin inhibitors (**p < 0.05).

It has already been shown that LMP and cathepsin D release might stimulate autophagy and induce changes in mitochondrial mass and membrane potential (ΔΨm). This might be a mechanistic explanation for the observed apoptotic phenotype. We therefore sought to further study the changes in selected autophagy markers (LC3I/II, ATG5 and Beclin-1) and mitochondrial alterations associated with the release of cathepsins (B, L and D) in the presence or absence of cathepsin inhibitors. Western blot analysis showed that the overexpression of PIG7 increased the expression of LC3I/II, ATG5, and Beclin-1 (Figure 7). Flow cytometry results showed that the overexpression of PIG7 had no significant effect on mitochondrial mass (*P > 0.05) (Figure 8A), but that it decreased the mitochondrial membrane potential (*P < 0.05) (Figure 8B). Finally, cathepsin inhibitors yielded resistance to the decrease in ΔΨm caused by the overexpression of PIG7 (**P < 0.05) (Figure 8).

**Figure 9:**
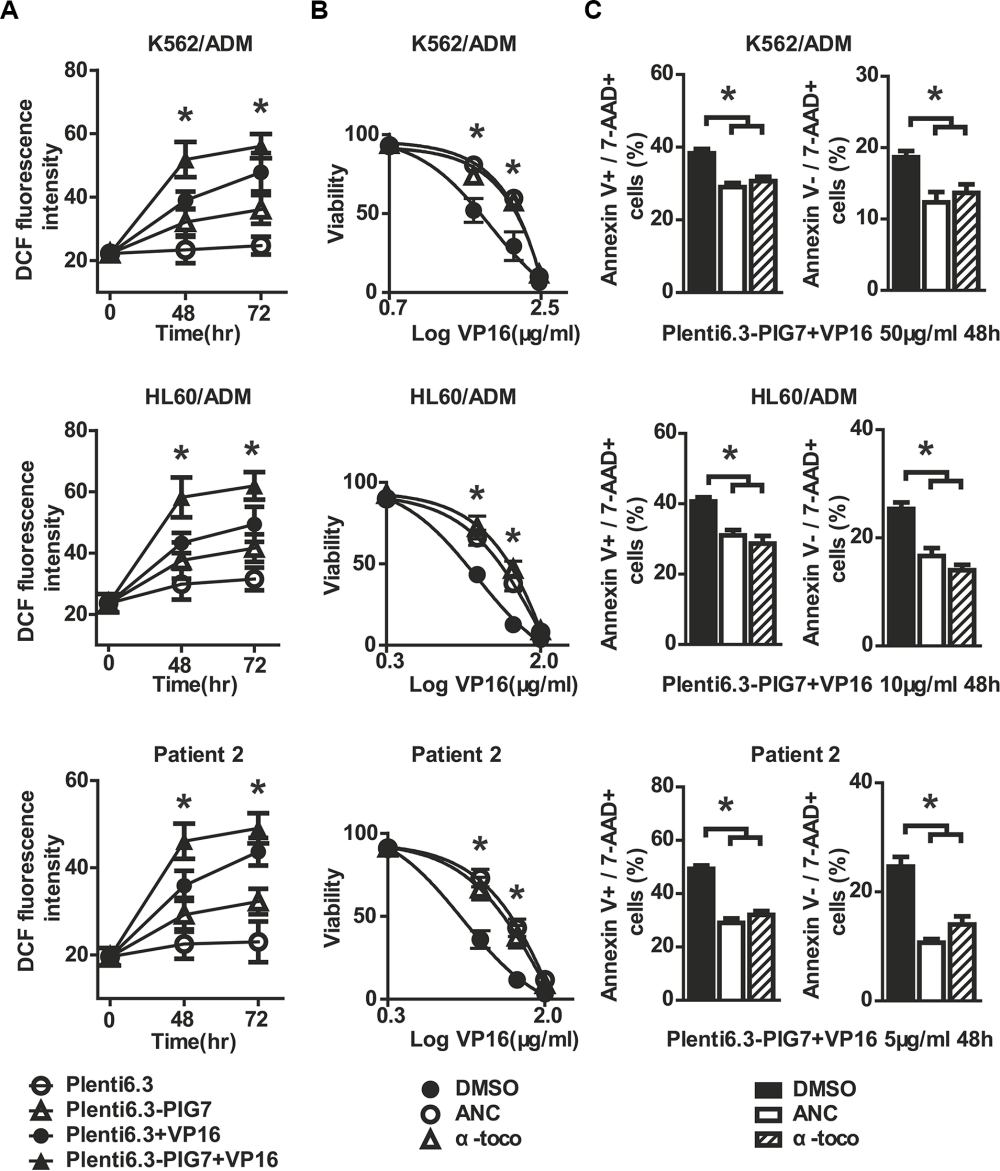
Antioxidants are protective against cellular toxicity (**A**) ROS detected by flow cytometry using DCFH-DA staining after lentiviral infection in combination with VP16. 48 and 72 h DCF fluorescence intensities both significantly increased in the Plent6.3-PIG7 group. The Plent6.3-PIG7 + VP16 group had the highest DCF fluorescence intensity (*P < 0.05). Viability and apoptosis/necroptosis rate of transfected cells following 48 h treatment with VP16 in the presence of α-toco or ANC. Data represent percent viability and apoptosis/necroptosis rate when compared to DMSO-treated cells. (**B**) MTT assay indicated that either α-toco or ANC increased the viability of leukemia cells in combination with Plent6.3-PIG7 and VP16 (*P < 0.05). (**C**) Annexin V staining also indicated that the two antioxidants protected leukemia cells from apoptosis and necroptosis (*P < 0.05).

Sufficient LMP leads to apoptosis by the release of cathepsins that allow for crosstalk with mitochondria as well as the release or stimulation of reactive oxygen species (ROS) [13]. To assess the involvement of ROS in the chemosensitive-promoting effects, the intracellular ROS levels were measured at 48 and 72 h after infection using an intracellular ROS assay kit (Cell Biolabs, San Diego, CA, USA). DCFH-DA is a cell-permeable, non-fluorescent probe that readily diffuses into cells, where it is oxidized by ROS to highly fluorescent DCF. As shown in Figure 5, overexpression of pig7 increased intracellular oxidative DCF fluorescence intensity, which is proportional to the amount of intracellular ROS. At 72 h, the DCF fluorescence intensity of K562/ADM, HL60/ADM, and patient 2 primary cells increased from approximately 24.7, 31.5, and 23.2 in the Plent6.3 groups to 36.1, 41.6, and 32.3, respectively. The combination of VP16 further increased ROS levels, such that the Plent6.3-PIG7 + VP16 group had the highest DCF fluorescence intensity (*P < 0.05) (Figure 9A). We then examined the impact of antioxidants on these cells and examined three groups, including an antioxidant α-tocopherol (α-toco), antioxidant n-acetylcysteine (NAC), and DMSO vehicle control. At 48 and 72 h after viral infection with Plenti6.3-PIG7, we found that with the decrease of ROS, both antioxidants increased leukemia cell viability (*P < 0.05) (Figure 9B). Annexin V staining also indicated that these two inhibitors had significant antagonistic effects on apoptosis and necroptosis (*P < 0.05 for both) (Figure 9C). These results suggest that ROS might contribute to the chemosensitivity-promoting effects of pig7.

As the caspase pathway has been extensively studied as an underlying mechanism for apoptosis [14, 15], we wished to examine whether it was involved in the chemosensitivity-promoting effects of pig7. Western blot analysis showed that the cleaved Caspase-9 and cleaved Caspase-3 proteins exhibited (i) low levels in both the Plent6.3-PIG7 experimental and Plent6.3 control groups and (ii) there was no difference between the two groups. Expression of Caspase 9/Apaf-1 complex was also unchanged (Figure 7). These results indicate that although overexpression of pig7 can induce LMP, decrease ΔΨm, and increase ROS, it has no effect on the activation of the caspase pathway.

**Figure 10:**
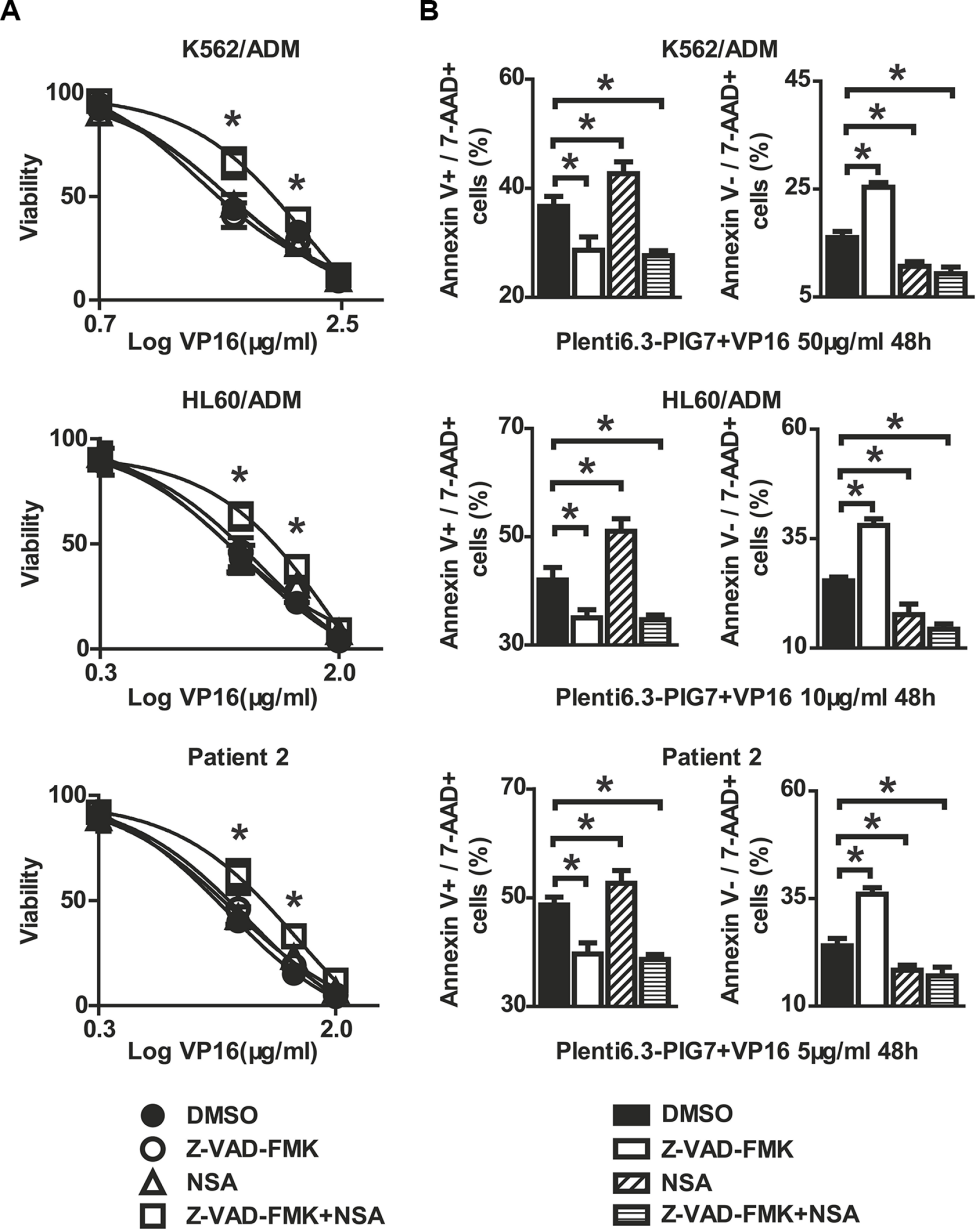
Protective effect of caspase inhibitor and necroptosis inhibitor on leukemia cells when combined with pig7 and VP16 Viability and apoptosis/necroptosis rate of transfected cells following 48 h treatment with VP16 in the presence of Z-VAD-FMK, Necrosulfonamide (NSA), or the combination of the two. Data represent percent viability and apoptosis/necroptosis rate when compared to DMSO-treated cells. (**A**) MTT assay indicated that neither Z-VAD-FMK nor NSA increased the viability of leukemia cells in combination with Plent6.3-PIG7 and VP16, but the combination of these two inhibitors can effectively protect these cells (*P < 0.05). (**B**) Annexin V staining indicated the cell apoptosis and necroptosis can be inhibited simultaneously when combined with Z-VAD-FMK and NSA (*P < 0.05).

We then examined the effect of the caspase inhibitor Z-VAD-FMK on subsequent changes in chemosensitivity using both a Z-VAD-FMK and DMSO control group. Interestingly, at 48 h post-infection with the Plenti6.3-PIG7 virus, we found that pretreatment with Z-VAD-FMK did not result in a protective effect for VP16 (Figure 10A). In addition, we examined the expression of a necroptosis marker, p-MLKL (phospho-mixed lineage kinase domain-like protein), and tested the protective effect of the necroptosis inhibitor necrosulfonamide (NSA) on these cells. Although Western blot analysis showed that p-MLKL exhibited higher levels in the Plent6.3-PIG7 group (Figure 7), our MTT assay revealed that NSA could not improve the viability of the transfected cells for VP16 (Figure 10A). Interestingly, when we combined Z-VAD-FMK and NSA, cell viability was significantly increased (*P < 0.05) (Figure 10A). Next, we analyzed the effect of these two types of inhibitors using an Annexin V staining assay. We found that (i) when Z-VAD-FMK was used alone, apoptosis was significantly inhibited (*P < 0.05) (Figure 10B), but the percentage of necroptotic cells was increased (*P < 0.05) (Figure 10B); (ii) when NSA was used alone, necroptosis was inhibited (*P < 0.05) (Figure 10B), but the percentage of apoptotic cells was increased (*P < 0.05) (Figure 10B); (iii) when combined with Z-VAD-FMK and NSA, cellular apoptosis and necroptosis were simultaneously inhibited (*P < 0.05) (Figure 10B).

### Cytosolic transfer of overexpressed PIG7 protein

**Figure 11:**
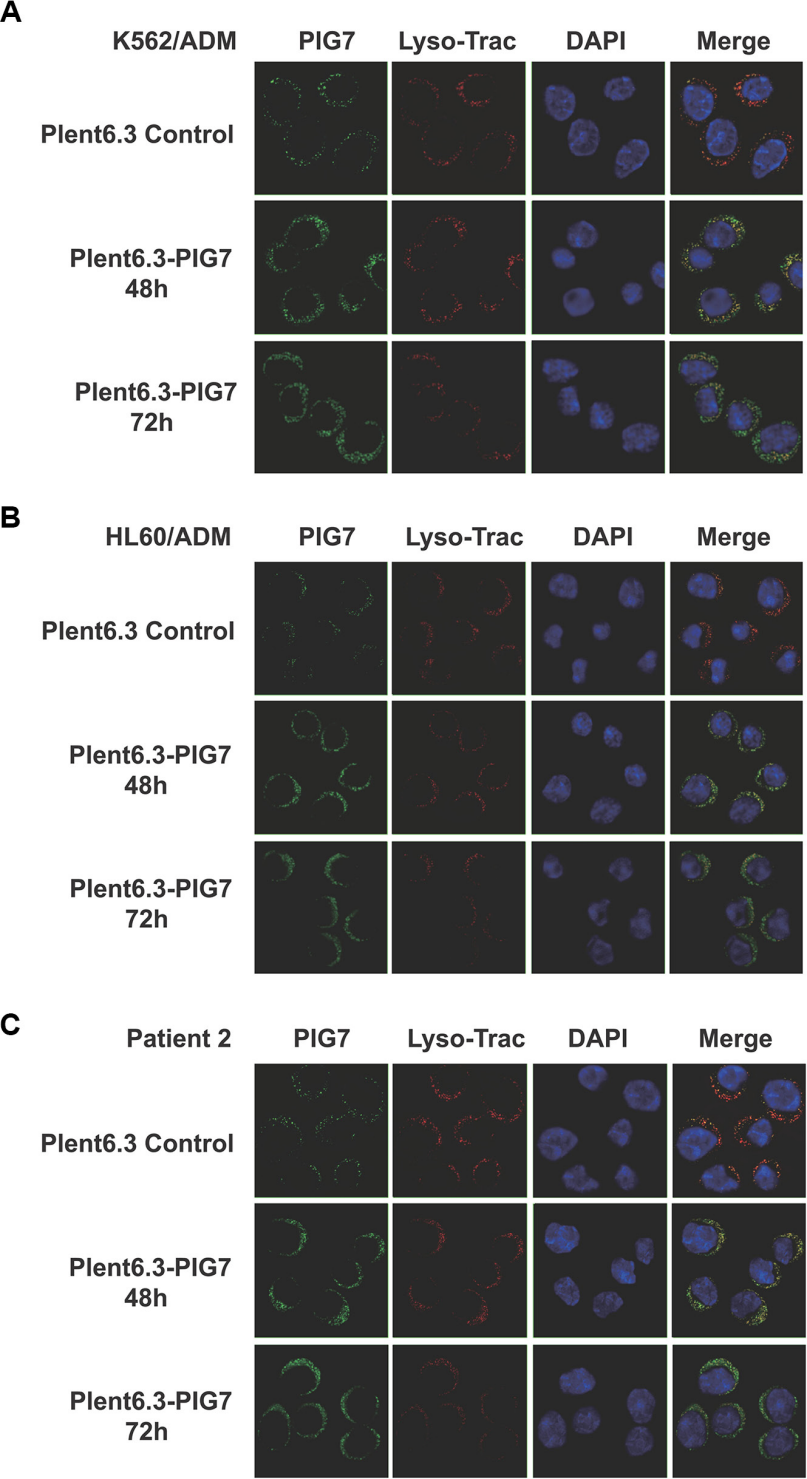
PIG7 and lysosomal cellular localization Live cells were incubated with LysoTracker red followed by fixation and permeabilization. Cells then underwent indirect immunofluorescence using anti-LITAF antibodies (green). The nuclei were labeled with DAPI (blue). At 48 and 72 h after infection with the Plenti6.3-PIG7 virus, green fluorescence was gradually enhanced, and a considerable number of green fluorescent signals appeared in the cytosol.

As mentioned above, PIG7 (SIMPLE) localized in lysosomal membrane in leukemia cells, but we found an interesting phenomenon that parts of PIG7 were transferred to the cytosol as pig7 expression increased. Western blot analysis showed that in the Plent6.3-PIG7 group, lysosomal and cytosolic expression of PIG7 were both significantly higher than expression in the Plent6.3 control group. The combination of VP16 and pig7 further improved the expression of cytosolic PIG7 (Figure 5). Confocal microscopy revealed that in the Plent 6.3 control group, the expression of PIG7 was relatively low and colocalized with LysoTracker Red. At 48 and 72 h post-infection with the Plenti6.3-PIG7 virus, we found that the expression of PIG7 was gradually enhanced. Moreover, that a considerable amount of PIG7 protein was transferred into the cytosol (Figure 11).

## DISCUSSION

Over the last decade, several lines of evidence have hinted at a possible role for PIG7 abnormalities in the pathogenesis of some malignancies, including downregulation in the urinary bladder and breast cancer carcinomas [16, 17] and mutation in Paget's disease [18]. Downregulated gene expression was also detected in methionine-induced apoptosis of melanoma cells [17]. PIG7 was silenced by homozygous deletion in primary mediastinal B-cell lymphoma and by promoter hypermethylation in germinal center lymphoma [19]. Our previous study has also revealed a striking decrease of PIG7 expression in newly diagnosed and refractory/relapsed acute leukemia patients. Further, these results were consistent with qRT-PCR performed in eight of ten leukemia cell lines as well as in acute leukemia patients who were in complete remission [6].

Many studies have shown that pig7 transcripts can inhibit tumor cell growth [8, 9]. The mechanism behind this effect can be attributed to PIG7 and its regulation of apoptosis. However, the universality of this effect needs to be considered in accordance with the specific cell line background. In our previous study, we found that ectopic expression of PIG7 in K562, HL60, NB4, and Raji cell lines was unable to affect differentiation status, proliferation potential, or induction of apoptosis. Interestingly, when combined with cytotoxic drugs such as VP16 and daunorubicin, a sensitizing effect could be observed. In this study, we further studied the relationship between PIG7 and this sensitization effect. We found that for different leukemia cells, the sensitizing effect of PIG7 was not only varied, but that it was related to the expression level of endogenous pig7. In other words, as the expression of endogenous pig7 was lower, the subsequent sensitizing effect was significantly higher. This pattern was evident for not only leukemia cell lines, but also primary cells.

As previously mentioned, PIG7 localized to the lysosomal membrane in leukemia cells, so we speculated that its chemosensitive-promoting effect might be associated with lysosomal membrane permeabilization (LMP). Accumulating evidence suggests that partial LMP with subsequent cytosolic release of lysosomal proteases may initiate and execute the apoptotic program in several models of apoptosis [20]. In some settings, apoptosis induction is dependent on an early release of cathepsins [21–26]. The mechanism underlying LMP is still incompletely understood. In this study, we found that although overexpression of pig7 significantly increased LMP, induced subsequent release of cathepsins, decreased the mitochondrial membrane potential, and activated autophagy, leukemia cells did not undergo apoptosis. We therefore speculated that there may be some intrinsic antagonism(s) preventing these apoptotic effectors from triggering apoptosis in leukemia cells. We then detected endogenous expression of some serine/cysteine protease inhibitors (Spi2A and Cystatin C) in the aforementioned cells after high PIG7 expression. Western blot analysis showed that Spi2A and Cystatin C proteins both exhibited higher levels in the Plent6.3-PIG7 experimental groups than Plent6.3 control groups (Figure 7). These intrinsic proteases inhibited lysosomal proteases (including the cathepsins B, D, L, G, H, K, and V) and protected cells from apoptosis. When combined with chemotherapy, LMP was further increased, with a larger amount of cathepsin released. Once this process reached the limits of cellular protection, this release became simultaneous with programmed cell death. Based on these results, we can infer that although the overexpression of pig7 resulted in LMP and subsequent cathepsin release, it was not enough to induce cellular death. The pig7-induced LMP can be thought of as an early event which pushed the leukemia cells to a “near apoptotic” state. This was also the same mechanism for pig7 to promote the chemosensitivity of leukemia cells.

A growing body of evidence suggests that the release of lysosomal proteases promotes apoptosis by acting on mitochondria to induce mitochondrial dysfunction directly or indirectly [13, 23, 27–33]. Addition of purified cathepsin B or D to mitochondria *in vitro* results in substantial ROS generation [31, 34]. Generation of ROS following mitochondrial dysfunction can feedback to the lysosome to maximize lysosomal permeabilization and, in turn, mitochondrial damage [35]. Cathepsin D can also trigger Bax activation and translocation to the mitochondria, where it induces the opening of pores on the outer mitochondrial membrane [32, 36]. In the present study, we found that during the induction of LMP (release of lysosomal proteases) by overexpression of pig7, the mitochondria membrane potential was decreased and the concentration of intracellular ROS was increased in parallel. Then we artificially added cathepsin inhibitors (E64D, CA-074Me and Pepstatin A) in above cells, and found these inhibitors were able to protect against the cytotoxicity of the combination of PIG7 with chemotherapy. Moreover, antioxidants (α-toco and NAC) could also play a similar protective effect. These results confirmed our conclusion from the other side.

For many years, caspases have been taken as the major class of proteases involved in the execution of cellular apoptosis [37–40]. There are a number of publications suggesting that caspases—under certain circumstances—are involved in the lysosomal release of apoptogenic factors [34, 41–46]. In our study, we found that the overexpression of pig7 alone could not activate caspase 9/Apaf-1 complex and caspase-3. Interestingly, the combination of pig7 and chemotherapy significantly increased the expression of cleaved capspase-9 and cleaved caspase-3, but applying the caspase inhibitor Z-VAD-FMK did not effectively protect leukemia cells. From the above results, we speculate that some caspase-independent cell death pathways such as necroptosis may be involved in this process, which was induced by the combination of pig7 and chemotherapy. We detected expression of necroptosis markers (RIPK1, RIPK3 and MLKL) and found overexpression of PIG7 can increase the expression of p-MLKL. However, we found that the necroptosis inhibitor NSA was unable to increase the viability of transfected cells with VP16 treatment. Interestingly, a combination of Z-VAD-FMK and NSA afforded a protective effect, with an Annexin V staining assay indicating that cellular apoptosis and necroptosis were simultaneously inhibited.

In conclusion, our findings implicate that the sensitizing effect of exogenous pig7 is more effective in leukemia cells which have lower endogenous pig7 expression. Increasing expression of pig7 can induce LMP and subsequent release of cathepsins and an increase in ROS production. Collectively, these place leukemia cells on the “verge of apoptosis”, therefore being more sensitive to cytotoxic drugs. Thus, caspase-dependent and caspase-independent pathways, such as necroptosis, are both involved in programmed cell death induced by combination of pig7 and chemotherapy. It is still unclear why PIG7 transfer to the cytosol during this process. It could be the result of a decreasing stability of the lysosomal membrane or the direct cause of LMP. These unanswered questions—along with whether or not other factors are also involved in the sensitizing process—remain unknown and ripe for further investigation.

## MATERIALS AND METHODS

### Construction of lentiviral vectors

PCR was used to amplify the fragment containing the PIG7 open reading frame from the pMD18T–PIG7 plasmid. Primer sequences were as follows: 5′-TGTAGGATCCGCCACCATGTCGGTTCCAGGACCT TAC-3′ and 5′-GTCAGCTAGCCTACAAACGCTTGTA GGTGCC-3′. The fragment was then subcloned into pLenti6.3-MCS/V5-DEST (Invitrogen, USA), which is referred to as “pLenti6.3-PIG7”. The empty vector pLenti6.3-MCS/V5-DEST was used in parallel as a control vector.

### Cell culture conditions

Two kinds of leukemia cell lines, K562 and HL60, were maintained in RPMI-1640 medium supplemented with 10% fetal bovine serum. The two drug resistant leukemia cell lines, K562/ADM and HL60/ADM, were maintained in RPMI-1640 medium supplemented with 10% fetal bovine serum and 1000 ng/ml doxorubicin.

Bone marrow samples were obtained from five AML patients admitted to the West China Hospital of Sichuan University. Ten healthy donors were used as controls. All patients and donors gave their informed consent for the study. Leukemia diagnoses and classifications were based on the criteria set forth in 2008 by the World Health Organization. Bone marrow mononuclear cells were cultured in Iscove's modified Dulbecco's medium supplemented with 15% fetal bovine serum for use in subsequent experiments.

### Lentivirus production and transduction

Lentiviral vector constructs were co-transfected into 293T cells with a packaging plasmid mix (Invitrogen, USA) using Lipofectamine 3000 transfection reagent (Invitrogen, USA). Infectious lentiviruses were collected at both 48 h and 72 h after transfection and were concentrated to 100-fold using ultracentrifugation. Leukemia cells were then transduced with the prepared lentivirus. After optimizing the conditions of lentiviral infection, our infection efficiency was found to be greater than 65% in each group. Resulting transgene expression was then detected by quantitative RT-PCR and Western blot.

### Quantitative real-time RT-PCR

Before and after lentiviral transfection, leukemia cells were collected and quantitative real-time RT-PCR (qRT-PCR) was performed according to our previous study [6]. The relative expression values were normalized to glyceraldehyde 3-phosphate dehydrogenase for cell lines or the median PIG7 expression of ten healthy donors for primary leukemia cells.

### Lysosomal isolation and enrichment

Leukemia cells were collected and the lysosomes were isolated using a Lysosome Enrichment Kit (Thermo Scientific, UT, USA) both before and after lentiviral transfection. Cells included those infected with the lentivirus alone or in combination with chemotherapy treatment. The isolated lysosomes and the non-lysosome-containing lysate were collected for subsequent Western blot analysis.

### Western blot analysis

The expression of PIG7, Pro-Cathepsin B, D, L, Cathepsin B, D, L, Caspase 3, 9, Cleaved Caspase 3, 9, LC3 I/II, ATG5, Beclin-1, Spi2A and Cystatin C were confirmed by Western blot both before and after leukemia cells were transfected with pLenti6.3-PIG7, or pLenti6.3 control vector (either alone or in combination with chemotherapy treatment). The immunoreactive proteins were visualized using the SuperSignal chemiluminescent detection system (Pierce, Rockford, IL, USA) in a similar exposure time. Antibodies against Caspase-3, Caspase-9, LC3B, ATG5, Beclin-1, Cystatin C and MLKL were purchased from Cell Signaling Technology (Danvers, MA, USA). Antibodies against Cathepsin B, Cathepsin D, Cathepsin L and β-actin were purchased from Santa Cruz Biotechnology (Dallas, Texas, USA). Antibody against PIG7 was purchased from BD Biosciences (San Diego, CA, USA). Antibody against Spi2A was purchased from Abcam (Cambridge, UK).

### MTT colorimetric assay

An MTT colorimetric assay was utilized to determine leukemia cells viability and sensitivity to chemotherapy treatment. Experiments were performed in triplicate and the resulting average used for all statistical analyses. The protocol used has been published previously by Morabito et al. [47]. The optical density (OD) of the cells was measured at 546 nm using a microplate reader (SLT-Lab, Salzburg, Austria). IC_50_ values were calculated using a linear regression that was determined between a series of drug concentrations and corresponding OD546 nm values measured by the MTT assay. The IC_50_ represented the drug concentrations required to achieve a 50% decrease in OD546 nm values.

### Apoptosis assessment by Annexin V staining

Leukemia cells were infected with lentivirus, and then parts of them were incubated with doxorubicin (ADM) or etoposide (VP16) of various concentrations respectively. The cells were collected at different time and analyzed for apoptosis by Annexin V-PE/7-AAD kit (BD Biosciences, San Diego, CA, USA) according to the manufacturer's instructions. All the samples were analyzed by FACScalibur flow cytometer (Becton Dickinson Immunocytometry Systems; San Jose, CA, USA).

### LMP assessment

AO staining analysis using flow cytometry: Cells were incubated with acridine orange (Sigma-Aldrich Corp. St. Louis, MO, USA) (1 μmol/L) for 30 minutes at 37°C. Cells were washed and their mean fluorescence intensity was quantified using a FACSCalibur flow cytometer (BD Biosciences, San Jose, CA).

AO staining analysis by fluorescence microscopy: Cells were incubated with acridine orange (1 μmol/L) for 30 minutes at 37°C. Cells were smeared with cytospin onto slides and immediately observed and imaged using an Olympus BX51 fluorescence microscope.

### Detection of reactive oxygen species (ROS)

Cells were incubated with DCFH-DA (Sigma-Aldrich Corp. St. Louis, MO, USA) (5 μmol/L) for 40 minutes at 37°C. Cells were washed and fluorescence intensity (Em 525 nm) was quantified with a FACSCalibur flow cytometer to determine the ROS levels.

### Detection of mitochondria mass and mitochondria membrane potential (ΔΨm)

Cells were incubated with MitoTracker Green FM (Invitrogen, Grand Island, NY, USA) (100 μmol/L) and MitoTracker Red CMXRos (Invitrogen, Grand Island, NY, USA) (100 μmol/L) respectively for 30 minutes at 37°C. Cells were washed and fluorescence intensity (Em 516 nm and Em 599 nm, respectively) was quantified with a FACSCalibur flow cytometer to determine the mitochondria mass and ΔΨm.

### Analysis of PIG7 expression and translocation by immunostaining

Cells were incubated with Lyso-Tracker Red (25 nM) for 30 minutes at 37°C. Cells were washed with PBS and smeared with cytospin onto slides. After permeabilization and blocking, slides were stained with an anti-PIG7 monoclonal antibody (BD Biosciences, San Diego, CA, USA). After applying a secondary FITC conjugated antibody, the nuclei were labeled with DAPI. PIG7 expression and translocation were then examined by Leica TCS Sp2 confocal microscopy (Leica Microsystems, Mannheim, Germany).

### Statistical analyses

All experiments were performed in triplicate. Statistical analyses and data plotting were conducted using GraphPad Prism 5 (GraphPad Software, San Diego, CA). Data represent the mean ± SD. Viability IC_50_ values at 48 h were calculated by line fitting normalized viability versus concentration with non-linear regression and statistical significance determined using one-way ANOVA. Differences in viability, caspase-3 activity, and apoptosis were analyzed using two-way ANOVA to identify differences. Any significant differences were confirmed with paired two-tailed *t*-tests. The significance of differences between two groups was determined using a paired *t*-test. A *p*-value of less than 0.05 was considered as statistically significant for all tests.
